# Textile-based supercapacitors for flexible and wearable electronic applications

**DOI:** 10.1038/s41598-020-70182-z

**Published:** 2020-08-06

**Authors:** Poonam Sundriyal, Shantanu Bhattacharya

**Affiliations:** 1 Microsystems Fabrication Laboratory, Department of Mechanical Engineering, Indian Institute of Technology, Kanpur, 208016 India; 2 Design Program, Indian Institute of Technology, Kanpur, 208016 India

**Keywords:** Energy storage, Supercapacitors, Batteries

## Abstract

Electronic textiles have garnered significant attention as smart technology for next-generation wearable electronic devices. The existing power sources lack compatibility with wearable devices due to their limited flexibility, high cost, and environment unfriendliness. In this work, we demonstrate bamboo fabric as a sustainable substrate for developing supercapacitor devices which can easily integrate to wearable electronics. The work demonstrates a replicable printing process wherein different metal oxide inks are directly printed over bamboo fabric substrates. The MnO_2_–NiCo_2_O_4_ is used as a positive electrode, rGO as a negative electrode, and LiCl/PVA gel as a solid-state electrolyte over the bamboo fabrics for the development of battery-supercapacitor hybrid device. The textile-based MnO_2_–NiCo_2_O_4_//rGO asymmetric supercapacitor displays excellent electrochemical performance with an overall high areal capacitance of 2.12 F/cm^2^ (1,766 F/g) at a current density of 2 mA/cm^2^, the excellent energy density of 37.8 mW/cm^3^, a maximum power density of 2,678.4 mW/cm^3^ and good cycle life. Notably, the supercapacitor maintains its electrochemical performance under different mechanical deformation conditions, demonstrating its excellent flexibility and high mechanical strength. The proposed strategy is beneficial for the development of sustainable electronic textiles for wearable electronic applications.

## Introduction

Smart electronic textiles have recently gained a lot of attention as a viable solution for the upcoming wearable electronics era^1,2^. The electronic textiles/ garments have demonstrated enormous potential in various applications such as; healthcare monitoring devices, environmental monitoring devices, military applications, entertainment, fashion technology etc^2–4^. Recently, the development of different textile-based electronic devices (like; wearable displays, wearable sensors, memory devices, and wearable transistors) have received greater attention from the scientific and engineering community, and their development is carried out on a rapid pace^4–6^. However, one of the major challenges for commercialization and further growth of wearable electronics is the lack of a compatible power supply that may possess the same level of flexibility, durability, weight, biocompatibility, and strength as the device itself^7,8^. The conventional energy storage devices fail to address these needs due to their rigid and bulky nature and also their inability to mount/perform on moving surfaces as is a critical need of wearable devices mounted on human and other beings^1,9–11^. Therefore, there exists a strong need for the development of flexible and high-performance energy storage devices which can be easily integrated with wearable electronics.

Among the various energy storage devices, thin and flexible supercapacitors are gaining more consideration for wearable electronics due to their salient features, such as excellent lifetime, lightweight, high power density, and their ability to deliver under mechanical deformation conditions^12–14^. However, their insufficient energy density still limits their use in practical applications. The energy density is directly proportional to the working potential and capacitance of the supercapacitor device^15^. Therefore, much research attention has focused on improving the potential range and energy density, either by using asymmetric cell assembly or by adopting the battery-supercapacitors hybrid electrode materials^16–18^. Recently, enormous efforts have been made for the realization of a hybrid combination of electric double layer capacitive (EDLC) material and pseudocapacitive/ battery-type material to combine the characteristic feature and potential ranges of both^17,19^.

Carbon-based materials such as activated carbon, graphene oxide and carbon nanotubes, are widely employed as EDLC materials due to their high surface area, good potential window, excellent rate capability and stable cycle performance^15,20^. On the other hand, the pseudocapacitive materials (such as RuO_2_ and MnO_2_) and battery-type materials (such as Ni(OH)_2_, Co_3_O_4_, NiCo_2_O_4_ etc.) are the potential Faradaic redox materials which can offer high capacitance/ capacity values, rich redox activity and higher electrochemical performance^15^. Recently, the binary and ternary composites of metal oxides have proved the remarkable electrochemical performance as compared to the individual materials resulting from the synergic effect among different components^21,22^. Among the several reported Faradaic redox materials, the ternary metal oxide composite of MnO_2_-NiCo_2_O_4_ has proved its strong potential for high energy density supercapacitors^21,23^. MnO_2_ and NiCo_2_O_4_ are well known pseudocapacitive and battery-like materials, respectively, which are extensively reported as excellent materials for energy storage applications^24^. However, the intrinsic low conductivity of the MnO_2_ and poor stability of the NiCo_2_O_4_ hinders their use for practical applications. Theoretically, the NiCo_2_O_4_ with high conductivity can act as a backbone to support and provide an effective electrical connection to the MnO_2,_ which improves the conductivity and active site density of the MnO_2_ component. It may remarkably boost the overall electrochemical performance of the composite. On the other hand, the presence of MnO_2_ ions augments the structural stability and voltage window of the NiCo_2_O_4_ material. Moreover, the MnO_2_ core-NiCo_2_O_4_ shell structure is one of the most promising designs for obtaining abundant electrochemical active sites. Enormous active sites produce numerous redox reactions and facilitate the fast diffusion of electrolyte ions, long cycle life and high charge storage capability as compared to their solitary parts.

Additionally, the selection of a suitable substrate with flexible and wearable features is a key requirement for the practical utilization of wearable supercapacitors^12^. Many efforts have been devoted to developing the fabric-based flexible supercapacitors for wearable applications^22,25,26^. So far, various fabric-based substrates, such as commercial carbon fabric^26^, polyester fabric^25^, cotton fabrics^22,27–29^, silk fibres^30^, and nylon lycra^31^ have been reported for wearable supercapacitors. However, the reported substrates still face the problem of low mechanical strength, high production cost, poor durability, and low conductivity. Therefore, it is essential to explore other textile fabrics that may overcome the existing problems of the wearable substrates. The bamboo fabric has recently emerged as an excellent choice for wearable clothing. Its various properties, such as excellent mechanical strength, lightweight, antibacterial properties, low cost, and high durability, make it suitable for wearable applications. Moreover, the bamboo plant is the fastest growing plant on earth which can replenish itself within a year and requires no water, fertilizers or pesticides to maintain its growth or quality; hence its harvesting cost is very low. Due to its beneficial features, it can serve as a potential fabric in wearable electronics applications for a sustainable future.

Efficient deposition of the energy storage materials over fabric substrates is another challenge to obtain good electrochemical performance and mechanical stability of the textile-based supercapacitors. Inkjet printing has recently emerged as an efficient tool for flexible electronics manufacturing^14,32^. It is a rapid and scalable method to transfer the device components over a variety of flexible substrates. Although the textile substrates are highly compatible with the inkjet printing process, some limitations of the inkjet printing process hinder its full utilization for practical applications. These limitations include the requirement of inks with rigid physical properties, the requirement of compatible post-treatment processes and size restriction of the ink particles^9,18,33^. The metal oxides are the promising energy storage materials and preparation of printable metal oxide inks with the required rheological properties pose a lot of difficulty due to their improper dispersion in most of the non-toxic solvents. During stable ink preparation, the presence of surfactants may also impede their electrochemical performance, and some defects may occur while reducing the particle size of the ink to meet the jet orifice criteria. The proper adhesion of printed material over the substrate is the other issue which may diminish the electrochemical performance of the device. Therefore, other strategies must be developed for smart deposition of the metal oxide components over the fabric substrates for wearable applications^34^.

**Figure 1:**
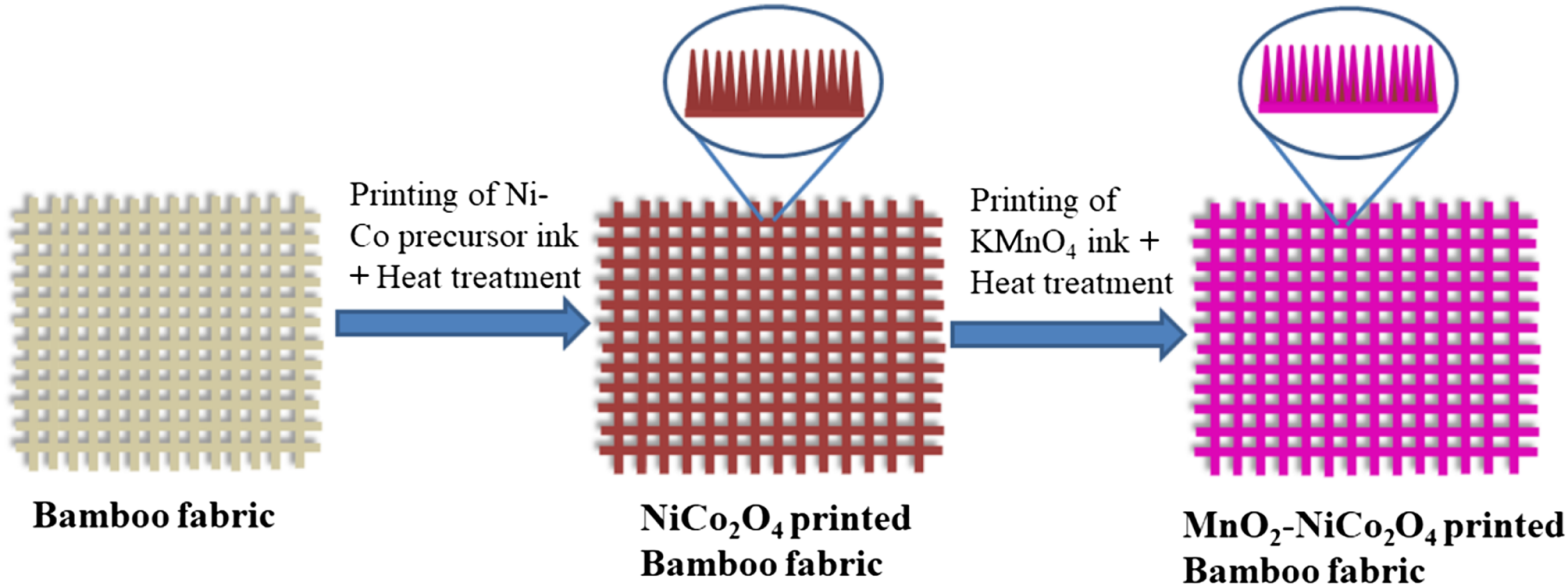
Schematic illustration of the fabrication process of printed MnO_2_–NiCo_2_O_4_ electrode over the bamboo fabric.

Here, inspired by the printing-assisted design of the textiles where patterns are stored in a soft format using motifs and printed by large scale printers using computer-aided design (CAD), we demonstrate the inkjet-printed MnO_2_–NiCo_2_O_4_//rGO asymmetric super-capacitors on the bamboo fabric textiles. Figure 1 provides the schematic illustration for the fabrication of metal oxide nanostructures over bamboo fabric through the inkjet printing process. To simplify the printing process, the metal precursors of MnO_2_ and NiCO_2_O_4_ have been directly printed over the fabric substrates. These were further processed at low temperatures to avoid fabric degradation. The printed rGO fabric and MnO_2_–NiCo_2_O_4_ fabric both show a high tensile strength of 442 MPa and 276 MPa, respectively. The developed MnO_2_–NiCo_2_O_4_//rGO printed asymmetric supercapacitors exhibited excellent electrochemical performance within a voltage range of 1.6 V. They have exhibited high areal capacitance of 2.12 F/cm^2^, the excellent energy density of 37.8 mW/cm^3^, a maximum power density of 2,678.4 mW/cm^3^, good cycle life and high rate capability. The high mechanical strength and excellent electrochemical performance of the printed bamboo textile-based supercapacitors demonstrate their potential as next-generation wearable electronics.

## Results and discussion

### Physical characterization and printing of inks

The physical properties of all the inks were carefully optimized to ensure the stable ink formation for a drop-on-demand printer. The viscosity and surface tension of water-based solutions were modified to the requirements of printable inks by using ethylene glycol and ethanol as detailed in our previous study^18^. The rheological properties of the inks were carefully optimized by controlling the Ohnesorge number (Oh)^33^. An Ohnesorge number is a dimensionless number which is related to the viscosity, surface tension, density and nozzle diameter of the printer as following:

1 $${\text{Oh}}=\frac{\sqrt{\text{Weber number}}}{\text{Reynolds number}}=\frac{\it \upeta }{\sqrt{a\uprho\upgamma }}$$

Here η, a, ⍴, and γ represents dynamic viscosity, characteristic length (diameter of the printing nozzle), density, and surface tension of the ink, respectively. The inverse of Ohnesorge number (Oh^−1^) is called Fromm number (Z).

All the inks were prepared in a solution of water, EG and ethanol with a ratio of 15:4:1 (water: EG: ethanol). The physical properties of the prepared inks are displayed in Table 1. According to Eq. (1), the calculated inverse Ohnesorge number for the different inks at a nozzle diameter of 20 μm comes out as 5.68, 5.94 and 5.52 for GO, Ni–Co precursor and KMnO_4_ precursor inks, respectively. These values are within the required range for printable inks (i.e. 4 < Oh^−1^ < 14). Another challenge for continuous and quality printing is to assure that the printer nozzle does not clog during printing. Therefore, all the inks were filtered using 450 nm filter to remove the large-sized particles. Figure S1 shows the particle size of all the inks based on DLS results which are within the required range. Therefore, the rheological properties and particle sizes of all the prepared inks are suitable for printing with a drop-on-demand printer.

**Table 1:** Physical properties of the inks (at 25 °C).

S. no	Ink composition	Density (Kg/m^3^)	Viscosity (mPa s)	Surface tension (mN/m)	Ohnesorge number(Oh)	Oh^−1^
1	GO	1,220	6.2	51	0.176	5.68
2	Ni–Co precursor	1,160	5.9	53	0.168	5.94
3	KMnO_4_	1,080	5.7	46	0.181	5.52

**Figure 2:**
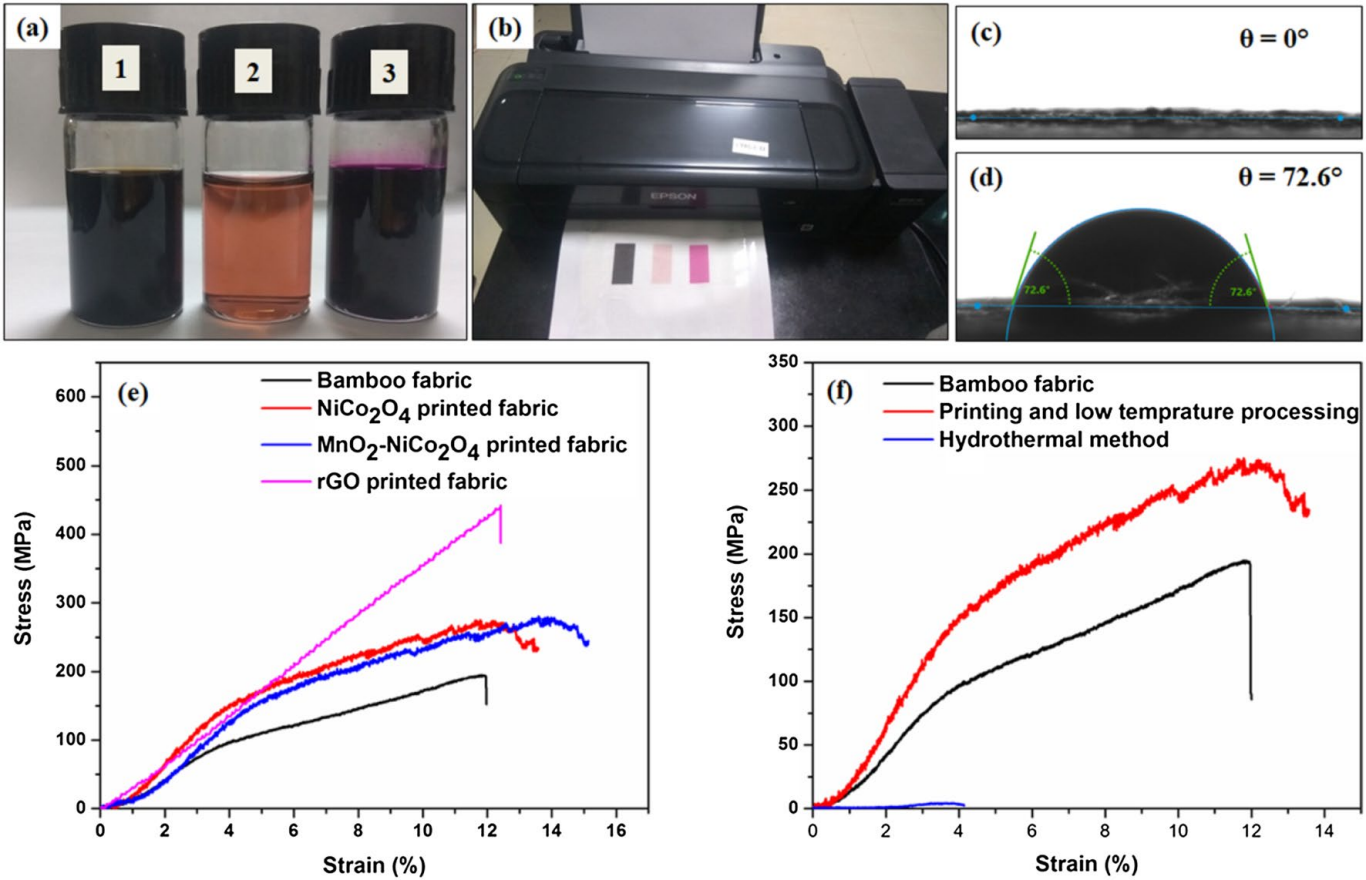
(**a**) Photograph of the inks (1: GO ink, 2: Ni–Co precursor ink, 3: KMnO_4_ ink), (**b**) printed inks on fabric substrates, (**c**) contact angle of the ink with untreated fabric substrate, (**d**) contact angle of the ink with PDMS coated fabric substrate, (**e**) comparison of stress–strain curves of different fabrics and (**f**) comparison of stress–strain curves of the NiCo_2_O_4_ fabrics developed with different methods.

All the different inks were filled in the ink cartridges of the EPSON printer and printing was performed in the low-speed and high-resolution mode of the printer. Figure 2a,b show the photographs of the prepared inks and printed patterns on the fabric substrate. Other than the optimization of the printing ink, the print quality depends on the surface of the substrate^35^. Figure 2c shows the ink contact angle with the fabric substrate, which is ~ zero due to the high absorption rate of the raw bamboo fabrics. The high spreading of the ink over the surface is undesirable to get a good quality of the print, which may also lead to material wastage. Therefore, the raw bamboo fabric is first coated with PDMS on the opposite side of the printing surface, which drastically improves the ink contact angle from 0 to 60° (Fig. 2d). Therefore, the bamboo fabrics with printing on one side and PDMS coating on the other side were used for further study. The printed rGO fabric and NiCo_2_O_4_ fabric exhibit low sheet resistances of 0.6 Ω and 0.4 Ω, respectively. Therefore, both the fabrics can act as a current collector as well as electrodes for the supercapacitor application.

The hydrothermal method with high-temperature sintering (> 180° C) of metal oxide is commonly used/ reported method to prepare the metal oxide-based electrodes. Despite its wide popularity, it is not suitable for the development of textile-based wearable devices due to its high-temperature requirement. Most of the wearable textiles are temperature sensitive, and they lose their mechanical strength at high temperature. Figure 2e shows a comparison of stress–strain profiles of the raw bamboo fabric, rGO printed fabric, NiCo_2_O_4_ printed fabric and MnO_2_–NiCo_2_O_4_ fabric. Tensile strength of the raw bamboo fabric is measured as 193 MPa, which was increased as 442, 271 and 276 MPa for rGO, NiCo_2_O_4_ and MnO_2_–NiCo_2_O_4_ printed fabrics, respectively. We have also prepared the NiCO_2_O_4_ fabric with the hydrothermal method, and the comparative stress–strain graphs are displayed in Fig. 2f. The hydrothermally grown NiCo_2_O_4_ over bamboo fabric has significantly lost its mechanical properties with a poor tensile strength of 3.66 MPa. Various cracks have also been observed in the FESEM images of the developed fabric, as shown in Fig. S2. It confirms the physical deterioration of the fabric due to hydrothermal treatment. Therefore, printing and low-temperature processing is a better option for the direct growth of metal oxide nanostructures over fabric substrates.

### Material characterization

**Figure 3:**
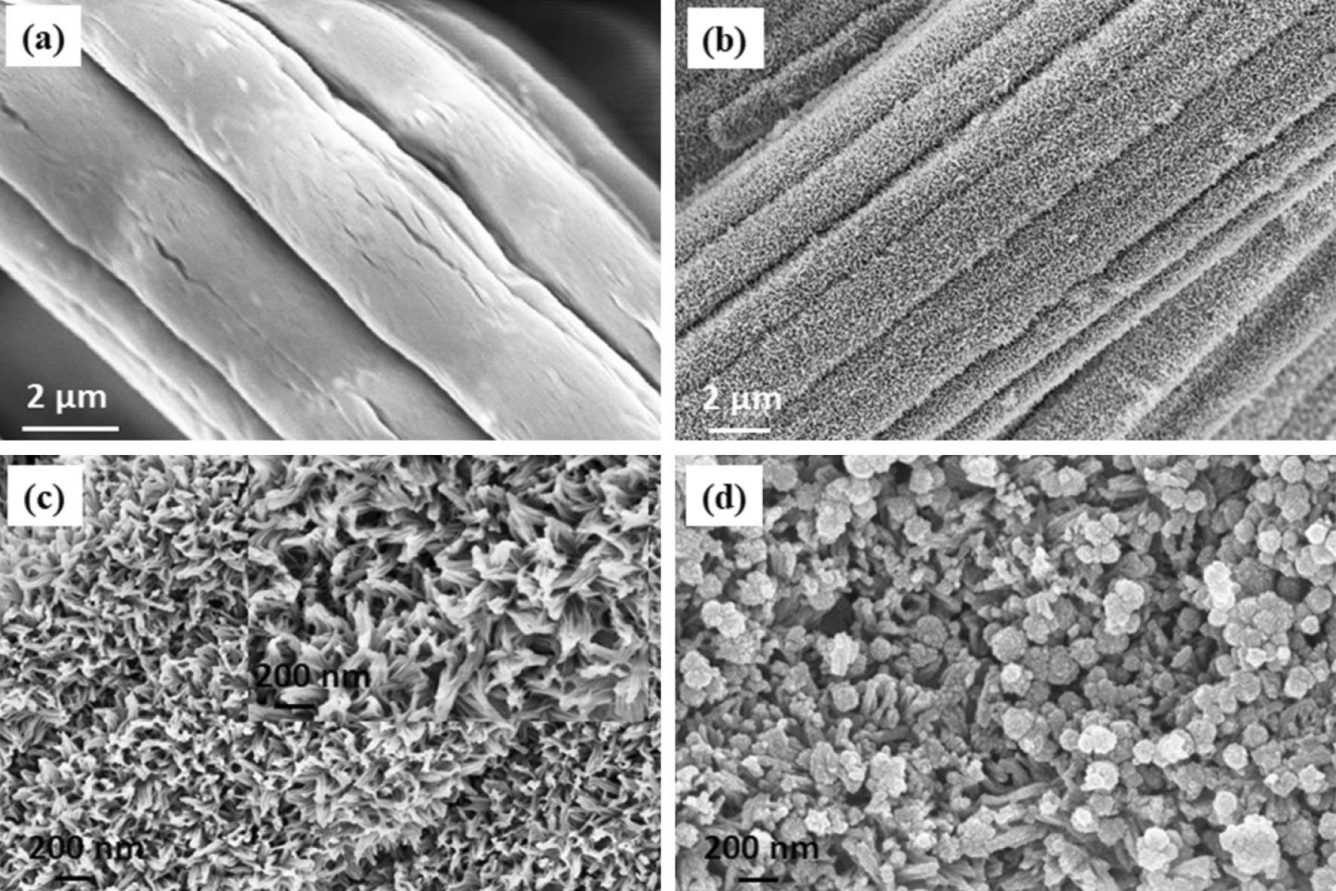
(**a**) The FESEM images of the Bamboo fabric, (**b**, **c**) low and high magnification SEM images of the NiCo_2_O_4_ printed bamboo fabric and (**d**) high magnification FESEM image of the MnO_2_–NiCo_2_O_4_ printed bamboo fabric.

FESEM and TEM were used to investigate the surface morphology and structure of the synthesized materials. Figure 3 shows the FESEM images of the fabric and the printed electrodes over it. Figure 3a,b displays the SEM image of raw bamboo fabric substrate and NiCo_2_O_4_ printed bamboo fabric, respectively. The NiCo_2_O_4_ printed bamboo fabric shows the dense and uniform NiCo_2_O_4_ growth over the fabric substrate. A high magnification SEM image is shown in Fig. 3c which further confirms the uniformity of the developed leaf-like nanostructures. The presence of open and free interspace in between the nanostructures is beneficial to provide the easy pathways for ion transport during the charge–discharge cycles. It will facilitate the easy accessibility of the electrolyte to the active electrode surface and will improve the electrode utilization. Figure 3d clearly indicates that MnO_2_ was deposited over the NiCo_2_O_4_ nanoleaves. The formation of this ternary composite is beneficial to improve the electrochemical performance of the supercapacitor device. The FESEM images of the rGO printed fabric also exhibit uniform printing of rGO layers over the fabric substrate (Fig. S3).

**Figure 4:**
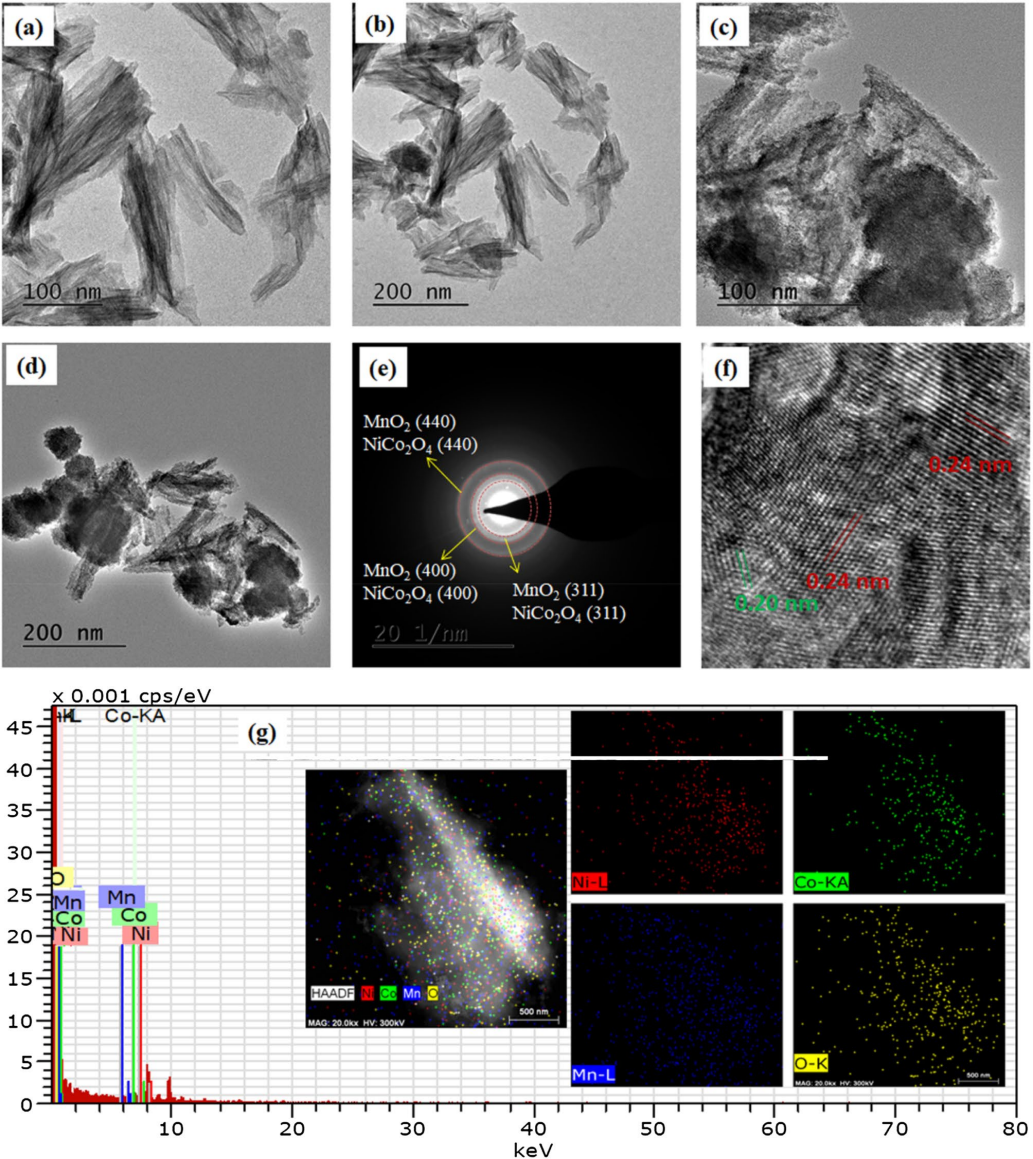
The TEM images of the: (**a**, **b**) NiCo_2_O_4_ and (**c**, **d**) MnO_2_–NiCo_2_O_4_ nanostructures; (**e**) SAED pattern of the MnO_2_–NiCo_2_O_4_ nanostructures, (**f**) HRTEM image of the MnO_2_–NiCo_2_O_4_ nanostructures and (**g**) EDS mapping of MnO_2_–NiCo_2_O_4_ nanostructures.

The detailed information about the surface morphology and structure was obtained by TEM. Figure 4a,b show the TEM images of the NiCo_2_O_4_ nano-leaves, which were similar to the FESEM results (Fig. 3c). Figure 4c,d clearly exhibit that the NiCo_2_O_4_ nano-leaves were uniformly wrapped by a layer of MnO_2_ which forms a core–shell like structure. The SAED ring pattern of MnO_2_–NiCo_2_O_4,_ as displayed in Fig. 4e indicate the reflections of (311), (400) and (400) crystallographic planes of both NiCo_2_O_4_ and MnO_2_. The high-resolution TEM (HRTEM) of MnO_2_–NiCo_2_O_4_ is shown in Fig. 4f. The lattice fringe with an interlayer spacing of 0.24 nm is related to the (311) plane of the MnO_2_ and NiCo_2_O_4_ and spacing of 0.20 nm is related to the (400) plane of NiCo_2_O_4_^36^. The EDS spectrum (Fig. 4g) and elemental mapping images (inset of Fig. 4g) further confirm the presence of Ni, Co, Mn and O in MnO_2_–NiCo_2_O_4_ composite. TEM image of rGO is displayed in Fig. S1 b which shows a layer like structure.

**Figure 5:**
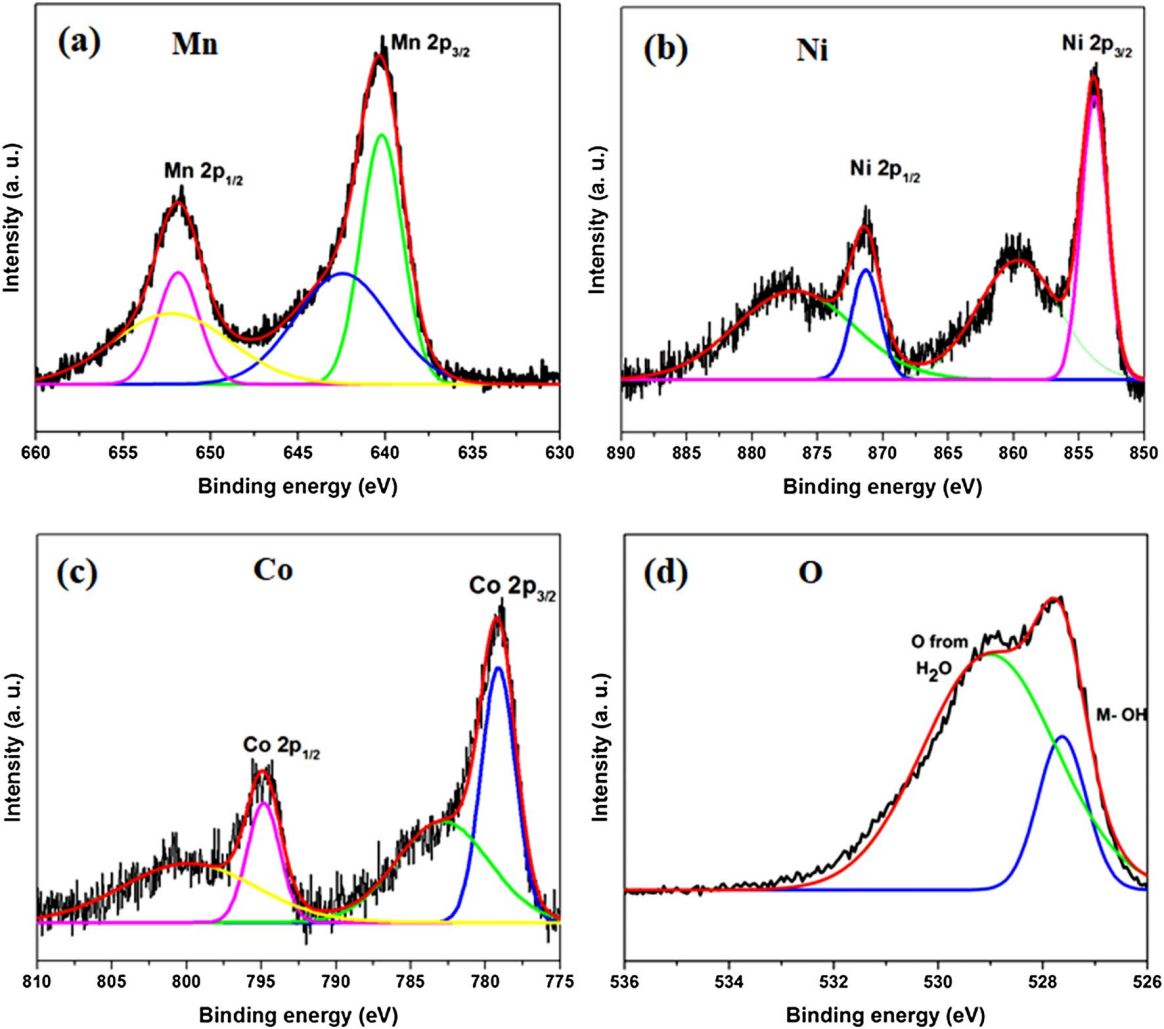
XPS survey scan of (**a**) Mn 2p, (**b**) Ni 2p, (**c**) Co 2p and (**d**) O 1 s, respectively.

The chemical state of the MnO_2_–NiCo_2_O_4_ composite was investigated by XPS spectrometer. It confirms the presence of Mn, Ni, Co and O elements in the nanocomposite. By using a Gaussian fitting method, the XPS spectrums of all elements were best fitted. The fitted XPS spectrum for Mn 2p is shown in Fig. 5a. The Mn 2p_3/2_ peak is centred at 640.3 eV, and Mn 2p_1/2_ peak is centred at 651.85 eV, with a binding energy separation of 11.55 eV. It reveals that the Mn species exists in the form of MnO_2_ in the composite and its binding energy separation is in good agreement with the previous literature^16,37^. The Ni 2p spectrum, as shown in Fig. 5b was characterized by two spin–orbit peaks at 853.75 eV (Ni 2p_3/2_) and 871.55 eV (Ni 2p_1/2_) with two satellite peaks, which indicates the existence of Ni^2+^ and Ni^3+^ in the composite^38,39^. The Co 2p XPS spectrum, as displayed in Fig. 5c was fitted with two spin–orbit peaks centred at 795 and 779 eV, respectively, which are characteristic of CO^2+^ and CO^3+^. The deconvoluted O1s peaks at 529.13 and 527.75 eV also confirm the presence of oxide in the composite (Fig. 5d). This result confirms the presence of MnO_2_ and NiCo_2_O_4_ in the composite and the successful formation of MnO_2_–NiCo_2_O_4_ composite as an electrode material^40,41^.

Figure S4a and b displays the XRD spectrum of the printed MnO_2_–NiCo_2_O_4_ composite and rGO over the bamboo fabric substrates. In Fig. S4a, a small peak at ~ 16° is related to the amorphous hemicellulose and lignin, and a peak at 22.7° is corresponding to the cellulose in the bamboo fabric. The major peaks located at 37°, 45°, 59° and 65° are related to the (311), (400), (511) and (440) crystallographic planes of both δ-MnO_2_ and cubic phase NiCo_2_O_4_ (JCPDS no. 042–1169 and 020–0781, respectively). These results are consistent with the HRTEM results. Figure S4b shows the rGO peaks at 26° and 44°, which are related (002) and (100) planes of carbon.

### Electrochemical analysis

#### Positive electrode

**Figure 6:**
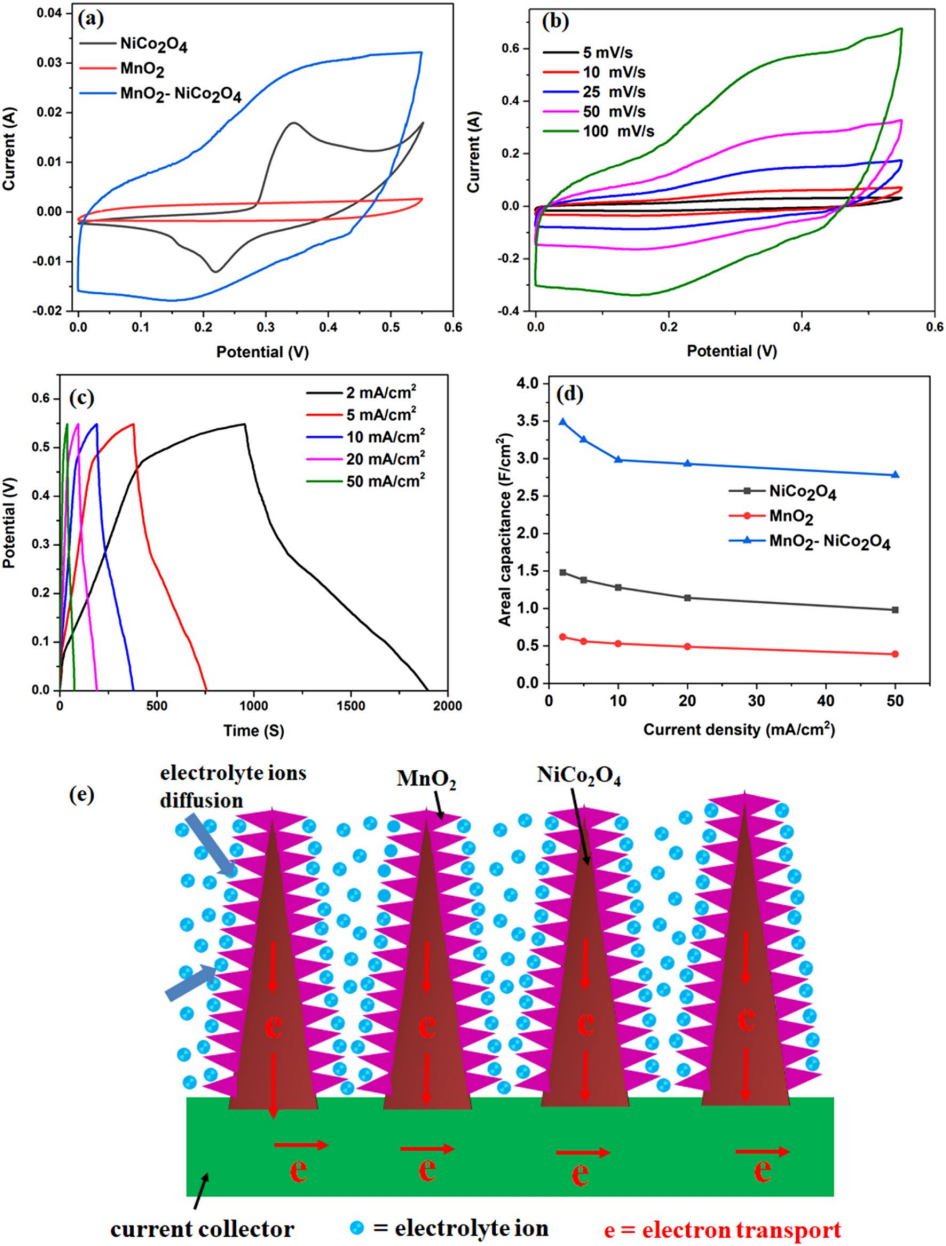
(**a**) Comparative CV curves of NiCo_2_O_4_ and MnO_2_–NiCo_2_O_4_ at a scan rate of 5 mV/s, (**b**) CV curves of printed MnO_2_–NiCo_2_O_4_ at different scan rates, (**c**) GCD curves of printed MnO_2_–NiCo_2_O_4_ at various current densities, (**d**) Comparison of areal capacitances of NiCo_2_O_4_ and MnO_2_–NiCo_2_O_4_ at different current densities, and (**e**) Schematic illustration of the electron and ion transport in the MnO_2_–NiCo_2_O_4_ electrode.

Figure 6 a shows the comparison of the CV curves obtained from the printed NiCo_2_O_4_ fabric, MnO_2_ fabric and MnO_2_–NiCo_2_O_4_ fabric at a scan rate of 5 mV/s. It clearly shows that the area integrated within the current-potential curve of the MnO_2_–NiCo_2_O_4_ fabric is much larger than the NiCo_2_O_4_ fabric, indicating better electrochemical activity and specific capacitance of the MnO_2_–NiCo_2_O_4_ fabric than NiCo_2_O_4_ fabric alone. It facilitates that the addition of MnO_2_ is beneficial to improve the electrochemical performance of the NiCo_2_O_4_ electrode. It may be due to the additional capacitance contributed by MnO_2_ shell that can adsorb Li^+^ cations on the electrode surface and/or intercalate and deintercalate Li^+^ ions^42^.

The CV curves of the NiCo_2_O_4_ fabric at different scan rates are shown in Fig. S5a. All the CV curves display a non-rectangular signature with sharp redox peaks indicating the behaviour of a pure battery-like electrode. While the CV curves if the MnO_2_ fabric exhibits a rectangular shape indicating supercapacitor like nature (as shown in Fig. S5b). Figure 6b shows the CV curves of MnO_2_–NiCo_2_O_4_ fabric which shows a combined signature of a battery-like and supercapacitor-like electrode. The presence of redox peaks in the form of a slight hump in all CV curves at around 0.35 V and 0.18 V could be attributed to the faradaic reactions related to the M–O/M–O–ON, where M represents Mn, Ni or Co and N represents electrolyte ion^37,43^. Presence of redox peaks in the CV curves of MnO_2_–NiCo_2_O_4_ fabric also confirms efficient utilization of the underlying NiCo_2_O_4_ core despite covered by the MnO_2_ shell. It indicates good accessibility of the electrolyte ions to both of the electrode materials.

Figure 6c shows the GCD curves of the MnO_2_–NiCo_2_O_4_ fabric electrode and Fig. S5c–d shows the comparative GCD profiles of the NiCo_2_O_4_ and MnO_2_ electrodes at different current densities. Similar to the CV results, the comparative GCD results show the better electrochemical performance of the MnO_2_–NiCo_2_O_4_ fabric electrode as compared to the NiCo_2_O_4_ and MnO_2_ electrode. The much longer discharge time of MnO_2_–NiCo_2_O_4_ fabric electrode as compared to the NiCo_2_O_4_ electrode (Fig. S6a) clearly indicates higher specific capacitance and high energy density of the MnO_2_–NiCo_2_O_4_ fabric electrode. The areal capacitances of the NiCo_2_O_4_, MnO_2_ and MnO_2_–NiCo_2_O_4_ fabric electrodes were calculated from their GCD curves based on Eq. (6), and the corresponding results are shown in Fig. 6d. The MnO_2_–NiCo_2_O_4_ fabric electrode possess the highest areal capacitance of 3.48 F/cm^2^ at 2 mA/cm^2^ current density, which is about 2.35 folds higher than the NiCo_2_O_4_ electrode (areal capacitance of 1.48 F/cm^2^) at the same current density. The areal capacitances for the MnO_2_–NiCo_2_O_4_ electrode at 5, 10, 20 and 50 mA/cm^2^ are found to be 3.25, 3.16, 2.92 and 2.83 F/cm^2^, respectively, which are about 2.3, 2.47, 2.56 and 2.82-times higher than the NiCo_2_O_4_ electrode. The areal capacitance values of MnO_2_–NiCo_2_O_4_ electrode were also greater than the MnO_2_ (~ areal capacitance values of 0.62, 0.56, 0.53, 0.49, 0.39 F/cm^2^ at current densities of 5, 10, 25, 50, and 100 mA/cm^2^, respectively). Additionally, the MnO_2_–NiCo_2_O_4_ electrode also exhibits a better rate capability with capacitance retention of 80% as compared to the NiCo_2_O_4_ and MnO_2_ electrodes which show poor rate capability of 66% and 63%, respectively, as the current density increases from 2 to 50 mA/cm^2^ (Fig. S6b). It confirms that the stability of the MnO_2_–NiCo_2_O_4_ electrodes are better than the NiCo_2_O_4_ electrode. The electrochemical performance of the developed MnO_2_–NiCo_2_O_4_ electrode was better than the available literature for the hybrid nanocomposites^16,21,23,40,44,45^.

Figure S7 shows the I–V plots and Nyquist plots of the MnO_2_ and MnO_2_–NiCo_2_O_4_ fabric electrodes depicting electrical resistances of these electrodes. Figure S7a shows the IV results of the electrodes using the four-point method. The MnO_2_–NiCo_2_O_4_ fabric shows much lower electrical resistance (~ 0.5 Ω-cm) as compared to the MnO_2_ electrode (~ 25 Ω-cm). Nyquist plots, as displayed in Fig. S7b further validates better conductivity of MnO_2_–NiCo_2_O_4_ fabric as compared to the MnO_2_ electrode. The measured series resistance for MnO_2_–NiCo_2_O_4_ fabric electrode was ~ 0.5 Ω, which was much lower than the series resistance of the MnO_2_ electrode. The IV and Nyquist results both confirm that the addition of NiCo_2_O_4_ has improved the electrical conductivity of the MnO_2_ electrode.

The excellent electrochemical performance of the MnO_2_–NiCo_2_O_4_ electrodes was due to the combined effect of the MnO_2_ and NiCo_2_O_4_ both. The electrochemical charge storage of the MnO_2_-based materials can be explained by the two mechanisms. One of these mechanisms involves intercalation/ deintercalation of the protons (H^+^) or cations (C^+^) in the bulk of the material. This mechanism is predominant in crystalline samples^14,37^.

2 $${\text{MnO}}_{{2}} + {\text{ C}}^{ + } + {\text{ e}}^{ - } \leftrightarrow {\text{MnOOC}}\;\left( {{\text{C}} = {\text{Li}},\;{\text{ Na}},\;{\text{ K}},{\text{ etc}}.} \right)$$

The second mechanism is based on the adsorption/ desorption of electrolyte cations (C^+^) on MnO_2_ surface. It is a surface process, which usually occurs in amorphous MnO_2_ samples^37^.

3 $$\left( {{\text{MnO}}_{2} } \right)_{{{\text{surface}}}} + {\text{ C}}^{ + } + {\text{ e}}^{ - } \leftrightarrow \left( {{\text{MnO}}_{2}^{ - } {\text{C}}} \right)_{{{\text{surface}}}} \;\left( {{\text{C}} = {\text{Li}},{\text{ Na}},{\text{ K}},{\text{ etc}}.} \right)$$

In case of NiCo_2_O_4_ material, the charge is stored on the surface and in bulk near the surface of the electrode^46^. It possesses a ferrimagnetic cubic spinal structure which has good electronic conductivity and multiple oxidation states. As reported in some of the literature^45,47^, the redox reactions in the alkaline electrolytes can be expressed as follows:

4 $${\text{NiCo}}_{2} {\text{O}}_{4} + {\text{OH}}^{ - } + {\text{H}}_{2} {\text{O}} \leftrightarrow {\text{NiOOH}} + {\text{2CoOOH }} + {\text{e}}^{ - }$$

5 $${\text{CoOOH}} + {\text{OH}}^{ - } \leftrightarrow {\text{CoO}}_{2} + {\text{H}}_{2} {\text{O}} + {\text{e}}^{ - }$$

During charging/ discharging process, there exist continuous valence states changes of Co^3+^/ Co^4+^ as well as M^2+^/M^3+^ (M = Co or Ni) on the electrode surface. It produces fast and reversible faradaic reactions^48^.

Figure 6e shows the schematic of electron and ion transport in the MnO_2_–NiCo_2_O_4_ electrode structure. The bamboo fabric provides porous support which ensures the fast and easy electrolyte ion transport and facilitates the full utilization of the active electrode surface. The improvement of the electrochemical performance may be attributed to the following points:

- All the electrodes were directly printed and synthesized over the bamboo fabric substrates that exclude the dead volume caused by the addition of polymer binders and conducting additives in the conventional processes.
- The NiCo_2_O_4_ has a very good electrical conductivity which provides the electron pathways for charge storage and delivery. It may act as efficient conducting support for the MnO_2_ material, which has a major disadvantage of poor conductivity.
- Both MnO_2_ and NiCo_2_O_4_ are excellent energy storage materials, and their combination will provide multifunctional and collective benefits.

#### Negative electrode

The rGO is a compatible material for the printed electronics and its high conductivity, large surface area, and good electrochemical properties are favourable for a negative electrode of an energy storage device. The CV and GCD curves of the rGO printed fabric are shown in Fig. S8 a and b, respectively. The CV profiles of the rGO at different scan rates exhibit a rectangular shape and GCD curves of rGO display a triangular shape with a linear discharge profile, which demonstrate the ideal capacitive nature of the negative electrode. According to Eq. (6), the maximum areal capacitances for negative electrode was calculated as 1.032 F/cm^2^ at a current density of 2 mA/cm^2^. It remained as 0.99 F/cm^2^ as the current density was increased upto 50 mA/cm^2^ indicating excellent rate capability (96%) of the selected negative electrode. Therefore, the use of rGO as a negative electrode may improve the rate capability and stability of the device.

#### Asymmetric device

**Figure 7:**
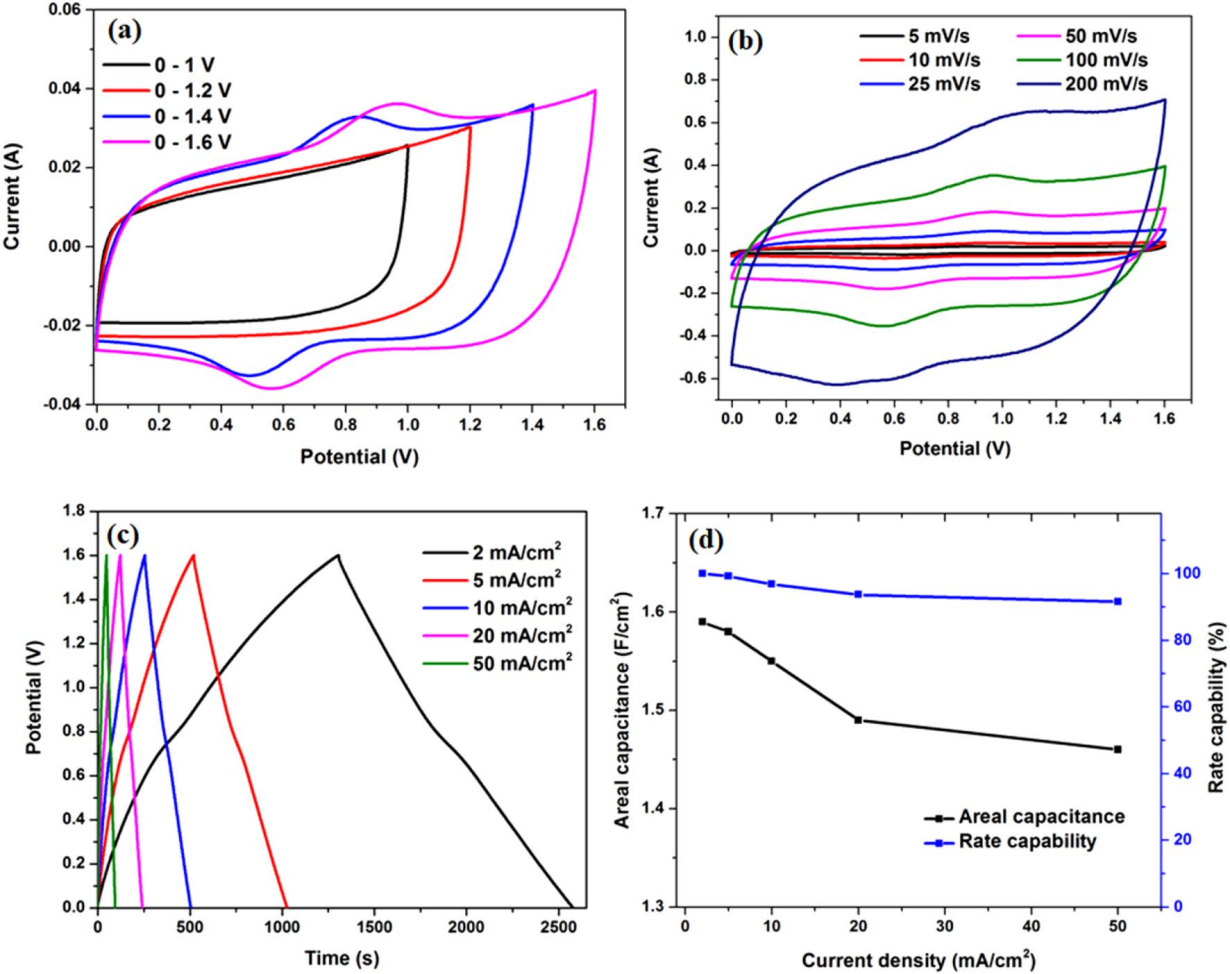
(**a**) CV curves of MnO_2_–NiCo_2_O_4_//rGO asymmetric device at different potential ranges at a scan rate of 10 mV/s, (**b**) CV curves of MnO_2_–NiCo_2_O_4_//rGO asymmetric device at different scan rates, (**c**) GCD profiles of the MnO_2_–NiCo_2_O_4_//rGO asymmetric device at various current densities and (**d**) The change in areal capacitance and rate capability of the MnO_2_–NiCo_2_O_4_//rGO asymmetric device as a function of current density.

Considering the excellent electrochemical performance of the MnO_2_–NiCo_2_O_4_ electrode and rGO electrodes in the 3-cell configuration, a 2-electrode device was assembled to further validate their practical application in supercapacitors. Based on Eqs. (12) and (13), the electric charge was balanced between the positive and negative electrodes, and the mass ratio of positive to negative electrodes was selected as 2:3. Fig. S8c shows the optimized CV curves of the positive and negative electrodes at a scan rate of 10 mV/s. The integral areas of two electrodes were almost similar within their corresponding voltage windows, which confirm a good charge balance between them. Taking the benefit of the dissimilar potential windows of the positive and negative electrodes, the total potential window of the device was extended upto 1.6 V. The problem of oxygen evolution reaction was observed on the positive electrode by further extending the potential range. Figure 7a shows the CV curves of the asymmetric supercapacitor device at different potential ranges from 1 to 1.6 V at a scan rate of 10 mV/s. According to Eq. (11), the areal capacitance increases from 1.576 to 2.53 F/cm^2^ as the potential was increased from 1 to 1.6 V. Accordingly, the energy density and output power can be improved by 311%. Therefore, the enlarged potential window will significantly improve the electrochemical performance of the supercapacitor device. Figure S8d exhibits the change of the areal capacitance of the asymmetric device with a variation in the potential window. A potential range of 0–1.6 V was used for further analysis of the asymmetric supercapacitor device.

Figure 7b shows the CV profiles of the MnO_2_–NiCo_2_O_4_//rGO asymmetric device at different scan rates within a potential window of 0–1.6 V. The CV profiles display a near-rectangular shape with the presence of redox peaks, which indicates the combined effect of the capacitive behaviour of the supercapacitor and redox nature of batteries. The obtained shape of the CV profile was retained even after applying a high scan rate of 200 mV/s, demonstrating the high rate capability of the device. The performance of the device was further characterized by GCD measurements. The GCD profiles of the device at current densities ranging from 2 to 50 mA/cm^2^ (Fig. 7c) displays near the rectangular shape with a slight variation from the linear profile of the discharge profile. This variation may be due to the presence of redox reactions arising from NiCo_2_O_4_ component of the positive electrode. The areal capacitance and rate capability of the device were calculated based on the GCD measurements and plotted versus current density (Fig. 7d). The device displayed a remarkable areal capacitance of 2.12 F/cm^2^ at a current density of 2 mA/cm^2^. Based on Eqs. (7) and (8), the specific capacitance and volumetric capacitance were calculated as 1766 F/g and 106 F/cm3, respectively. When the current density increases from 2 to 5, 10, 20 and 50 mA/cm^2^, the areal capacitance was reduced to 2.1, 2.06, 1.99 and 1.95 F/cm^2^, respectively [specific capacitance of 1,750, 1716, 1658, 1625 F/g, respectively and volumetric capacitance of 105, 103, 99.5, 97.5 F/cm^3^, respectively]. Accordingly, the device displayed excellent rate capability of 92% as the current density was changed from 2 to 50 mA/cm^2^ (Fig. 7d).

**Figure 8:**
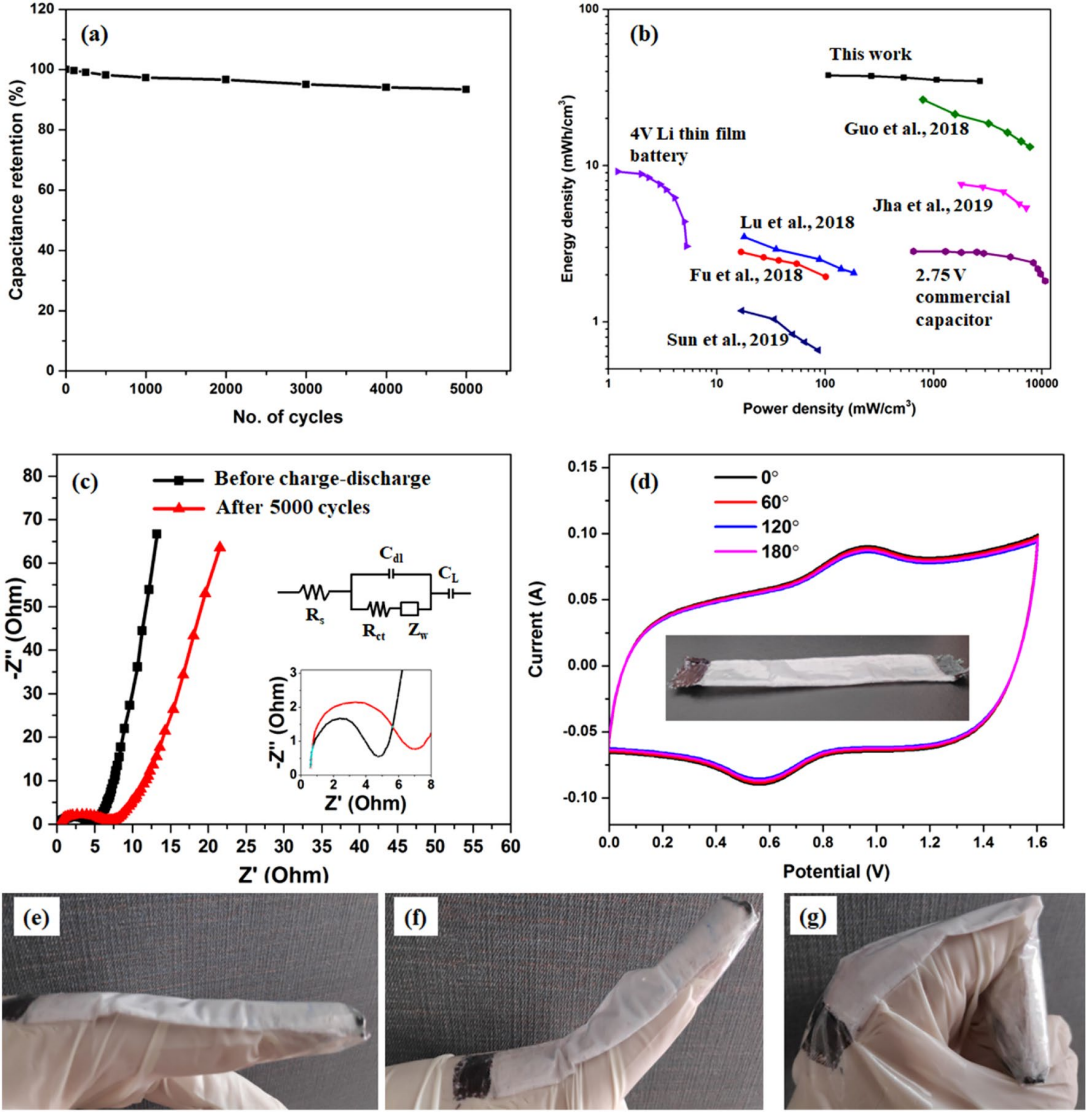
(**a**) Capacitance retention of the device with the different number of charge–discharge cycles, (**b**) Ragone plot showing comparison of energy and power densities of our device with existing literature, (**c**) Nyquist plot of the device before and after cycle life test, (**d**) CV curves of the device under different mechanical bending conditions (inset image shows digital photograph of textile supercapacitor), and (**e**–**g**) Photographs of the wearable supercapacitor device under different finger moving conditions.

The device also exhibited excellent capacitance retention of 93.1% even after 5,000 charge–discharge cycles at a current density of 50 mA/cm^2^ (Fig. 8a). This result confirms the high stability of the device and facilitates its use for real-life applications. Figure 8b shows the Ragone plot of the assembled bamboo fabric-based asymmetric supercapacitor device based on the GCD results in a potential range of 0–1.6 V. Data of some relevant studies are also plotted for comparing our device performance with the existing literature. According to Eqs. (9) and (10), the assembled device can deliver a maximum energy density of 37.8 mWh/cm^3^ at a power density of 107.06 mW/cm^3^ and retains 34.67 mWh/cm^3^ at a power density of 2,678.36 mW/cm^3^. The obtained combinations for energy and power density are higher than the existing literature for wearable supercapacitors^25,27,49–53^.

The EIS test was further conducted to investigate the stability of the device before and after 5,000 charge–discharge cycles, and the recorded Nyquist plot within a frequency range of 0.1 Hz to 100 kHz is shown in Fig. 8c. An equivalent circuit, as shown in the inset of Fig. 8c was used to fit the impedance data. R_s_ is related to the combination of ionic resistance of the electrolyte, contact resistance of the electrode/ current collector interface and resistance of the substrate. This R_s_ value was obtained from the intercept of the real part in the high-frequency region. The charge transfer resistance at the electrode/electrolyte interface is denoted by R_ct_, which is represented by the semicircle in the high-frequency range. The R_ct_ indicates the presence of Faradaic reactions and double layer capacitance in the cell^15^. The R_s_ value was almost identical before and after cycles, i.e. 0.59 and 0.61 Ω, respectively. The R_ct_ value has increased from 3.2 to 4.89 Ω after 5,000 cycles. The minor deviation of the resistance values even after 5,000 cycles indicates good stability of the asymmetric device. The achieved EIS results are comparable to the available literature in the area of fabric-based energy storage devices. The flexibility is an important requirement for wearable applications^54–56^. To investigate the mechanical flexibility of the device, the device was bent at different bending angles, and the corresponding variation in electrochemical performance is measured using the CV test. Figure 8d shows CV curves of the device at a scan rate of 25 mV/s at different bending angles. All the curves almost overlapped with each other, indicating no structural failure and capacitance loss under different mechanical deformation conditions. Figure 8e–g also displays the photographs of the textile supercapacitor device under different motions of a finger.

The study of the self-discharge of flexible supercapacitors has great significance in practical applications^57,58^. However, the self-discharge characteristic of the supercapacitors is frequently neglected in previous research. Figure S9 shows the self-discharging profile of the developed MnO_2_–NiCo_2_O_4_//rGO fabric supercapacitor device under open-circuit conditions for 12 h after being charged at 1.6 V. The supercapacitor displays a rapid self-discharge rate for the initial time which gradually slows down after some time. The open-circuit voltage of the device was constant (~ 1.24) after 12 h, which indicates low self-discharge characteristic of the device. The low self-discharge characteristic of the device implies its significance for its application for wearable electronics.

## Methods

### Preparation of negative electrode ink

The GO was synthesized from the graphite flakes using Modified Hummers method. The GO ink was prepared by exfoliating GO in water. Typically, the GO was sonicated for 1 h under ultrasonication bath and then centrifuged for 10 min at 2000 rpm. The EG and ethanol were properly mixed in the prepared solution (solution: EG: ethanol = 15: 4: 1) to optimize the physical properties of the inks. This ink was used to print the negative electrode of the asymmetric device.

### Preparation of positive electrode inks

The inks for positive electrodes were prepared by the precursors of Ni, Co and Mn. Briefly, 10 mM Ni(NO_3_)_2_·6H_2_O, 20 mM Co(NO_3_)_2_·6H_2_O and 15 mM urea were dissolved in 60 mL of deionized water under magnetic stirring. Further, 16 mL of EG and 4 mL of ethanol were mixed in the prepared solution to adjust the physical properties of the ink. The prepared ink was used to print the NiCo_2_O_4_ component/ core of the positive electrode. The MnO_2_ component of the positive electrode was prepared from KMnO_4_. Typically, 3 mM KMnO_4_ was dissolved in 60 mL of water using magnetic stirring. Further, EG and 4 mL of ethanol were mixed in the prepared solution to adjust the physical properties of the ink.

### Printing

All the components were printed over the bamboo fabric substrate using EPSON L130 desktop printer. The rheological properties of the inks were carefully optimized by controlling the Ohnesorge number (Oh)^33^. All the inks were filtered using a 450 nm syringe filter to remove any large-sized particles. After optimizing the Ohnesorge number, all the inks were filled in the separate ink cartridges of the printer. Printing was performed in the high –quality and low-speed mode of the EPSON printer to get the maximum drop volume of the inks. The fabric substrate was pasted on A4 paper to ensure the proper movement of the fabric over the sheet feeder of the printer. The GO ink was printed over the fabric substrate and subsequently reduced with hydrazine hydrate to form the negative electrode. The Ni and Co precursor ink was printed on a different location of the fabric. It acts as a seed layer over the fabrics. The Ni and Co printed fabrics were treated at 80° C for 6 h in a refluxed system by keeping them upside down in the precursor solution. The prepared fabrics were cleaned with water and further dried at 80° C for 24 h in an oven followed by irradiation treatment using a flash lamp. It has developed the NiCo_2_O_4_ nanostructures over the fabric substrate. The KMnO_4_ precursor ink was reprinted over the already developed NiCo_2_O_4_ nanostructures, and the printed fabrics were again treated at 80° C for 6 h. The as-synthesized MnO_2_—NiCo_2_O_4_ fabric acts as a positive electrode for the asymmetric supercapacitor device.

### Material characterization

The viscosities of the inks were determined by a rheometer (Anton Paar) while surface tension and contact angle were measured by a contact-angle goniometer. The particle sizes of the inks were determined by dynamic light scattering (DLS; Beckman Coulter Delsa Nano C). The crystal structures and morphologies of the printed materials were characterized by X-ray diffraction (XRD; PANalytical diffractometer with Cu Kα radiation), X-ray photoelectron spectrometer (XPS, PHI5000VersaProbe), field emission scanning electron microscopy (FESEM; Zeiss Supra 40VP) and transmission electron microscopy (TEM; 300 kV) equipped with an energy dispersive X-ray spectrometer (EDX). The tensile strength of the printed fabrics was determined by Universal Testing Machine (Instron-1195).

### Electrochemical characterization

All the electrochemical characterizations, including cyclic voltammetry (CV), galvanostatic charge–discharge (GCD) and electrochemical impedance spectroscopy (EIS) were performed on potentiostat/ galvanostat (Autolab 302 N). Initially, the individual electrodes were investigated in three-electrode mode using inkjet-printed MnO_2_–NiCo_2_O_4_ fabric, NiCo_2_O_4_ fabric and rGO fabric as working electrodes, 6 M LiCl as an electrolyte, Pt wire as a counter electrode and Ag/ AgCl (3 M KCl) as a reference electrode. The electrochemical performance of the device was investigated by using MnO_2_-NiCo_2_O_4_ fabric as the positive electrode, rGO fabric as a negative electrode, PVA–LiCl gel as a solid-state electrolyte and bamboo fabric as a separator. The as-prepared electrodes were used for the characterization without using any additives or binders, which could significantly reduce the total dead area in the electrode. It will be beneficial to improve the electrolyte – active electrode interaction due to easy electrolyte ion migration. The areal capacitance (C_a_) of the electrode samples, and the device was evaluated from GCD curves by the following equation:

6 $$\text{C}_{\rm a}=\frac{\text{i}\Delta \text{t}}{ \text{a}\Delta \text{V} }$$

The gravimetric/specific capacitance (Cs), volumetric capacitance (C_V_), energy density (E_V_) and power density (P_V_) of the flexible supercapacitors were calculated from the GCD curves according to the equations as follows:

7 $$Cs=\frac{i\Delta t}{m\Delta V}$$

8 $$\text{C}_{\rm v}=\frac{\text{i}\Delta \text{t}}{\text{V}\Delta \text{V}}$$

9 $$\text{E}_\text{v}=\frac{1}{2}\text{C}_{\rm v}\Delta \text{V}^2$$

10 $$\text{P}_{v}=\frac{\text{E}_{\rm v}}{\Delta \text{t}}$$

Here, i is the constant charge/discharge current, $\Delta$ t is the discharge time, a is the surface area of the electrode, V is the total volume of the asymmetric supercapacitor and $\Delta$ V is the applied potential window.

The areal capacitance (C) is calculated from the CV curves according to the following equation:

11 $$\text{C}=\frac{\text{A}}{2\text{sa}\Delta \text{V}}$$

where A is the total integrated area under the CV curve, s is the scan rate, a is the area of the electrode surface, and △V is the applied voltage window.

For an asymmetric supercapacitor, it is essential to balance the stored electric charge between positive and negative electrodes (q_+_  = q_−_). The stored electric charge depends on capacitance (C), voltage range ($\Delta$ V) and mass loading (m) according to the following relation:

12 $$\text{q}=\text{m}\times \text{C}\times \Delta \text{V}$$

13 $$\frac{\text{m}_+}{\text{m}_-} =\frac{\text{C}_- \times \Delta \text{V}_-}{\text{C}_+ \times \Delta \text{V}_+}$$

Here, m_+_ and m_−_ are masses of active materials on positive and negative electrodes, respectively.

### Fabrication of asymmetric device

The electric charge was balanced between MnO_2_–NiCo_2_O_4_ fabric (positive electrode) and rGO fabric (negative electrode) before assembling them for asymmetric supercapacitor. The PVA–LiCl gel electrolyte was applied over both the electrodes and bamboo fabric separator. The asymmetric device was fabricated by assembling both electrodes with the fabric separator in between. The whole assembly was heat-treated in an oven for 20 h at 40° C. The solidified device was encapsulated by parafilm foil and PDMS to avoid any moisture absorption. The total mass of the electrode was ~ 1.2 mg.

## Conclusion

In summary, we have successfully developed a precursor-based approach for the growth of MnO_2_–NiCo_2_O_4_ metal oxide structures over bamboo fabric substrates by using the inkjet printing technique. This simple method allows us to fabricate the large-area, high density and patterned metal oxide structures over fabric substrates in a low-cost, environment-benign and time-efficient way. The MnO_2_–NiCo_2_O_4_ printed bamboo fabric was used as a positive electrode, and rGO printed bamboo fabric was used as the negative electrode of the asymmetric supercapacitor device. The assembled MnO_2_–NiCo_2_O_4_//rGO device displays stable performance within a potential range of 0–1.6 V and demonstrates excellent electrochemical performance including high capacitance of 2.12 F/cm^2^, the high energy density of 37.8 mW/cm^3^, a maximum power density of 2,678.4 mW/cm^3^, reasonable cycle life with 92% of capacitance retention after 5,000 cycles and low charge-transfer resistance of 3.2 Ω. Thus, precursor-based printing may be a feasible solution for the development of wearable devices on the textile substrates. The current approach with ease of fabrication, environment sustainability and low manufacturing cost hold great potential for the upcoming wearable electronics era.

## Supplementary information

Supplementary file1
