# Supplementary material for: Textile-based supercapacitors for flexible and wearable electronic applications
Source: Sci Rep. 2020 Aug 6;10:13259. doi: 10.1038/s41598-020-70182-z (PMC7411075; doi:10.1038/s41598-020-70182-z)
Supplement: Supplementary file 1 — Supplementary file1 [file 41598_2020_70182_MOESM1_ESM.pdf]

Supplementary Information

Textile-based supercapacitors for flexible and wearable electronic applications

Poonam Sundriyal[a], and Shantanu Bhattacharya[a,b,*]

b. Microsystems Fabrication Laboratory, Department of Mechanical Engineering, Indian Institute of Technology, Kanpur, India, 208016

b. Design Program, Indian Institute of Technology, Kanpur, India, 208016

[*]Corresponding author's Email Id: bhattacs@iitk.ac.in

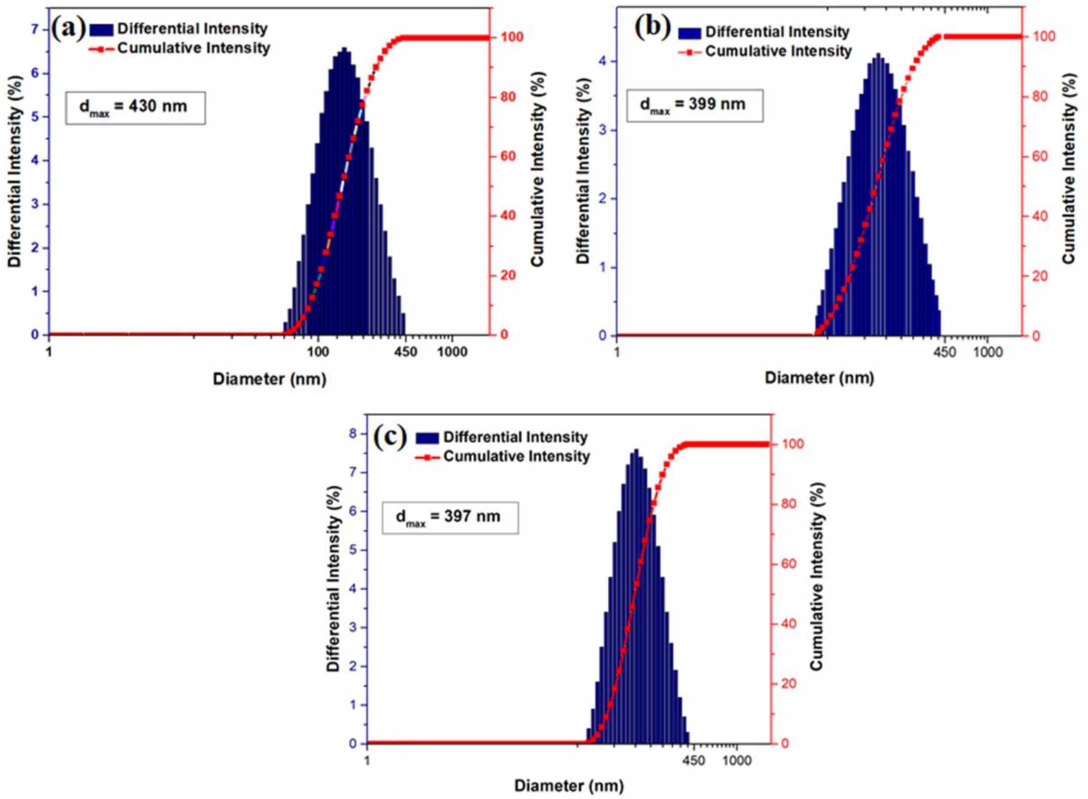

Figure S1. DLS results showing particle sizes of: (a) rGO ink, (b) Ni- Co precursor ink and (c) $KMnO_4$ precursor ink.

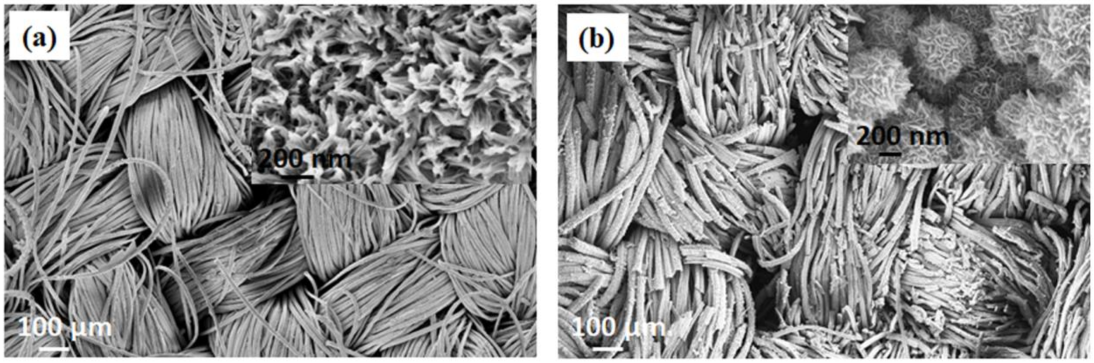

Figure S2. FESEM results of $NiCo_2O_4$ developed over bamboo fabric with: (a) printing and low-temperature processing method, and (b) hydrothermal method.

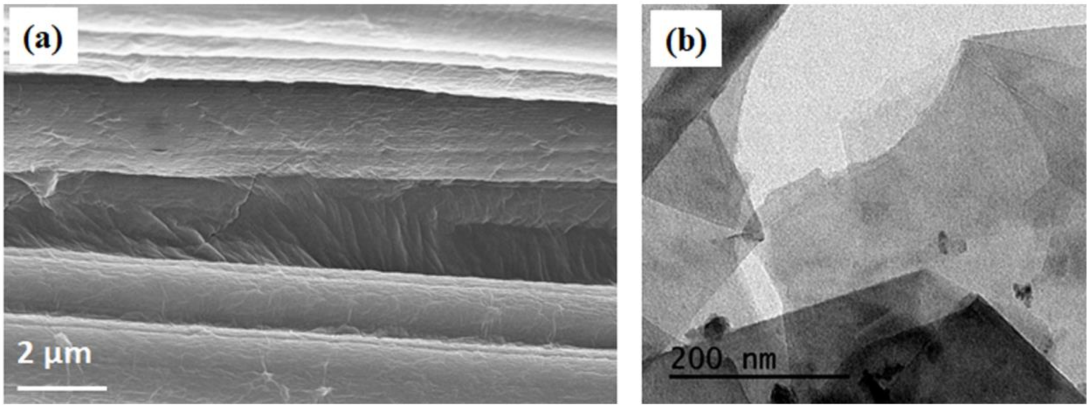

Figure S3. (a) The FESEM images and (b) TEM image of rGO printed bamboo fabric.

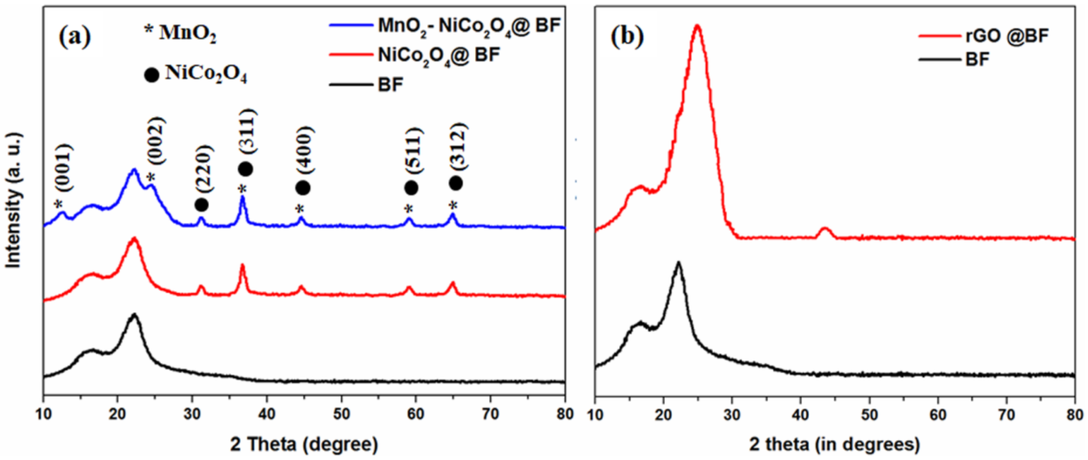

Figure S4. XRD patterns of: (a) Bamboo fabric, $NiCo_2O_4$ printed bamboo fabric and MnO_2-$NiCo_2O_4$ printed bamboo fabric and (b) rGO printed bamboo fabric.

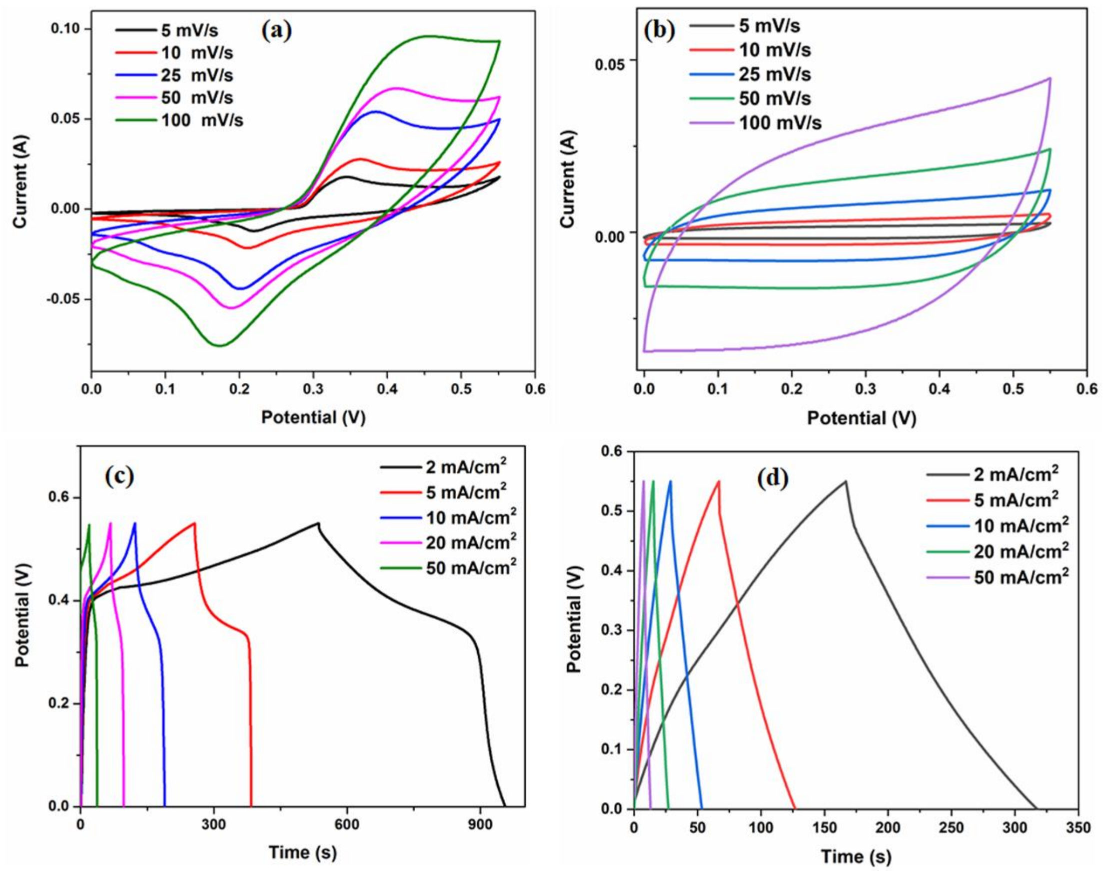

Figure S5. (a) CV curves of the $NiCo_2O_4$ printed bamboo fabric at different scan rates, (b) CV curves of the MnO_2 at different scan rates, (c) GCD curves of the $NiCo_2O_4$ printed bamboo fabric at different current densities, and (d) GCD curves of the MnO_2 at different current densities.

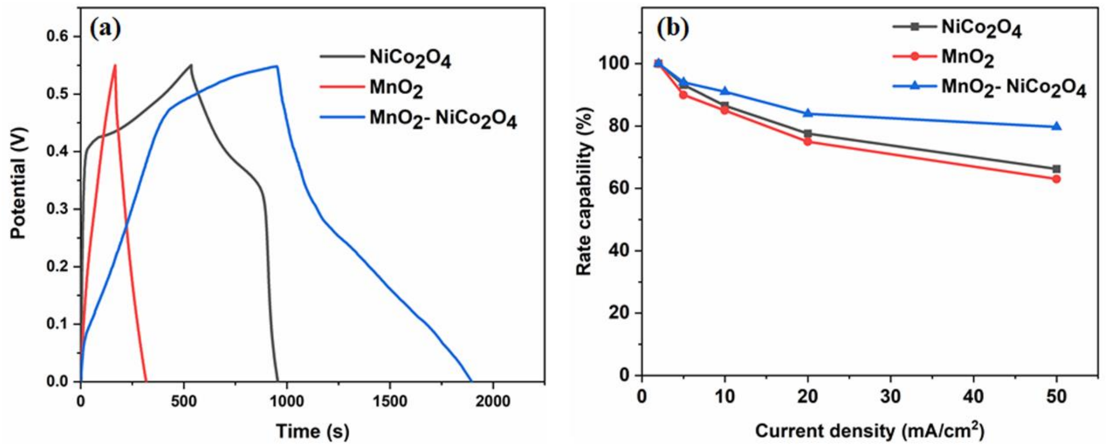

Figure S6. (a) Comparative GCD curves of $NiCo_2O_4$, MnO_2 and MnO_2- $NiCo_2O_4$ printed fabric at a current density of 2 mA/cm^2, and (d) Comparison of the rate capability of the $NiCo_2O_4$ and MnO_2- $NiCo_2O_4$ printed fabric at different current densities.

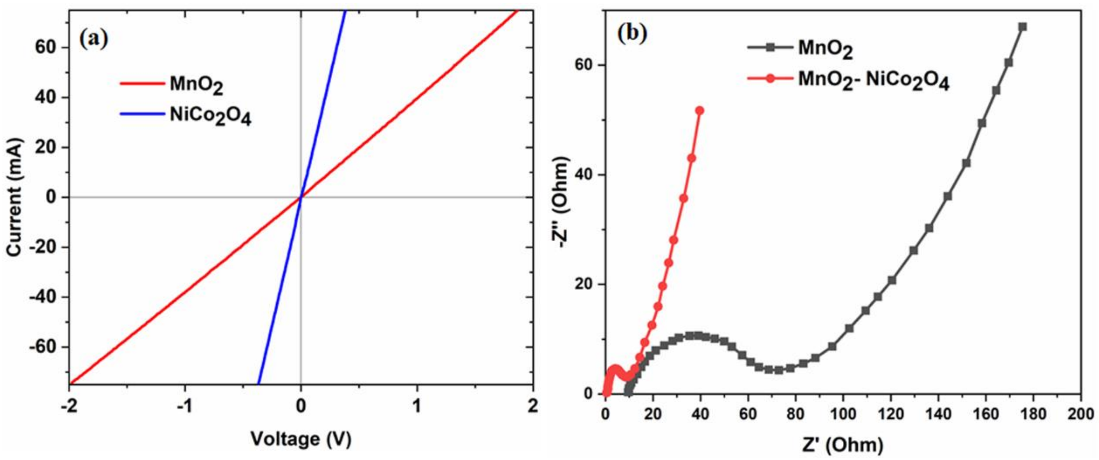

Figure S7. (a) I-V plots and (b) Nyquist plots of the MnO_2 and MnO_2- $NiCo_2O_4$ fabric electrode.

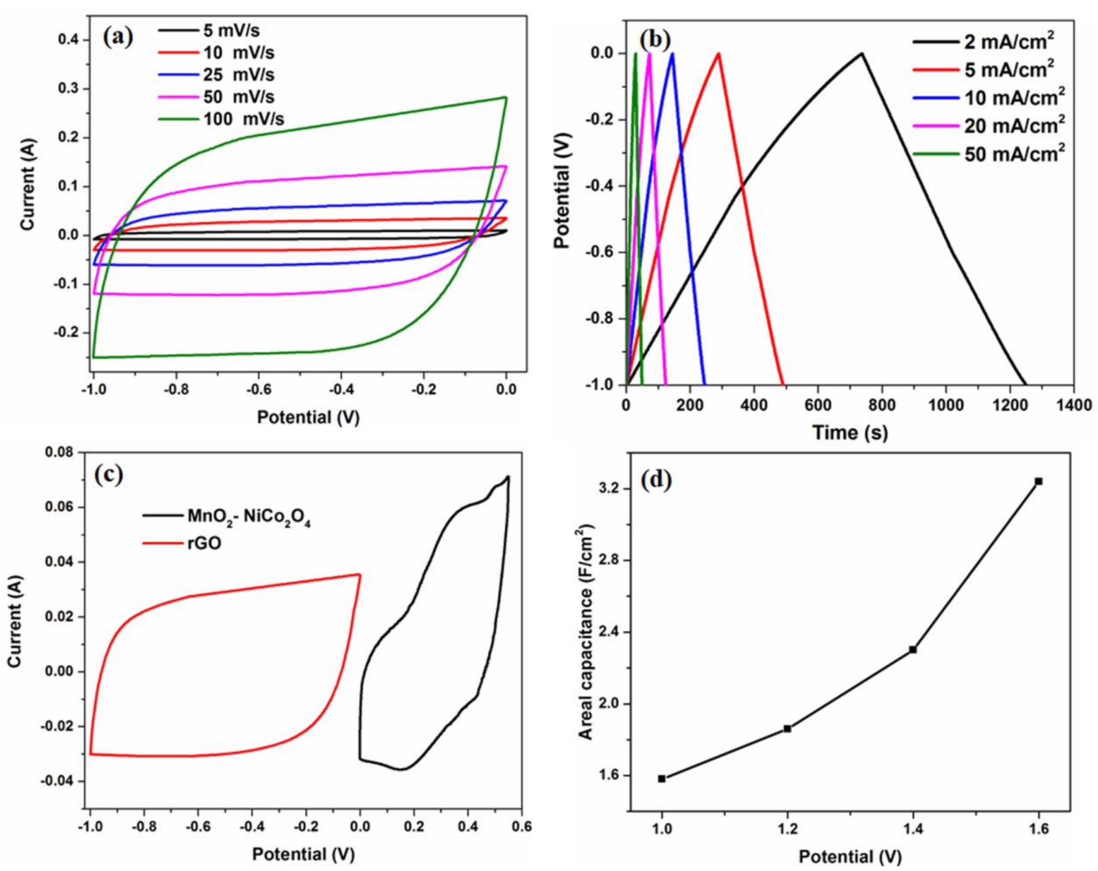

Figure S8. (a) CV curves of the rGO printed bamboo fabric at different scan rates, (b) GCD curves of the rGO printed bamboo fabric at different current densities, (c) CV curves of the rGO and MnO_2- $NiCo_2O_4$ printed fabric at 10 mV/s scan rate, and (d) Variation in the areal capacitance of the rGO// MnO_2- $NiCo_2O_4$ asymmetric device at different potential ranges.

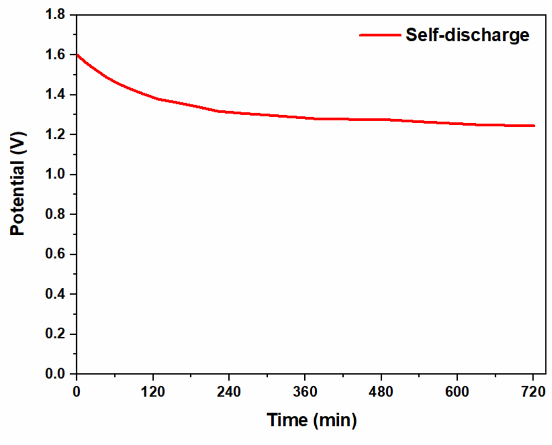

Figure S9. Self-discharge curve of the MnO_2- $NiCo_2O_4$ // rGO fabric supercapacitor device under open-circuit conditions for 12 h after being charged at 1.6 V.
